# Adverse Fetal Outcomes and Histopathology of Placentas Affected by COVID-19: A Report of Four Cases

**DOI:** 10.7759/cureus.44402

**Published:** 2023-08-30

**Authors:** Megan Corn, Truc Pham, Walter Kemp

**Affiliations:** 1 Obstetrics and Gynaecology, University of North Dakota School of Medicine and Health Sciences, Grand Forks, USA; 2 Pathology, Incyte Pathology, Spokane Valley, USA; 3 Pathology, State of Montana, Forensic Science Division, Billings, USA

**Keywords:** prenatal transmission, coronavirus disease 2019 (covid-19), sars-cov-2, case series, chorionic histiocytic intervillositis, fetal demise, placenta

## Abstract

Coronavirus disease 2019 (COVID-19) has had significant impacts on mothers and neonates. In this report, we present four unique cases of COVID-19 infections in pregnancy and its effects on the mother, fetus, and placenta. Four mothers presented to the hospital during their pregnancy. Each had tested COVID-19-positive 1-29 days prior to admission. Gestational age ranged from 16 weeks six days to 36 weeks six days. Three of the four cases resulted in fetal demise or infant expiration. The common finding among all four cases was pathologic changes in the placenta. Most of the placentas were small for gestational age and had extensive villous infarction. There was also histiocytic intervillositis with villous necrosis and perivillous fibrin deposition. The placentas demonstrated positive staining of syncytiotrophoblasts for severe acute respiratory syndrome coronavirus 2 (SARS-CoV-2) spike S1 subunit protein. SARS-CoV-2 RNA was detected in tissue samples of two of the fetuses demonstrating vertical transmission. A higher incidence of severe COVID-19 disease course has been observed in pregnant women. Prior to the SARS-CoV-2 pandemic, chorionic histiocytic intervillositis of the placenta was rarely seen, and mostly of unknown etiology. The increase in placental fibrin levels results in decreased maternal placenta blood flow ensuing hypoxic stress in the fetus. Intrauterine hypoxia has been associated with alterations in brain structure and function resulting in defects in motor skills, cerebral palsy, decreased brain weight, schizophrenia, and other forms of cognitive impairment.

## Introduction

The appearance of severe acute respiratory syndrome coronavirus 2 (SARS-CoV-2) resulted in the worldwide pandemic that was declared on March 11, 2020 [1]. The first case was identified in the Hubei province of China in December 2019 and the virus has since mutated many times [2]. The delta variant (B.1.617) emerged in India in December 2020 and by May 2021, had been detected in 43 countries [3]. It was estimated to be more than two times as contagious as previous variants of the disease and may cause a more severe illness [4]. The newest variant of SARS-CoV-2, B.1.1.529 Omicron, was reported to the World Health Organization (WHO) on November 24, 2021. As of August 16, 2023, there have been 769,806,130 confirmed cases and 6,955,497 deaths worldwide [5].

SARS-CoV-2 is highly contagious and has high rates of transmission [6]. There is variability in the presentation of the disease. Most commonly the presenting symptoms include cough, fever, shortness of breath, malaise, and loss of taste and/or smell, but some patients may progress to a more severe illness or even death [7]. There was an increase in maternal mortality by 33.3% after March 2020, corresponding with the onset of coronavirus disease 2019 (COVID-19) [8]. In addition to the horizontal transmission of the SARS-CoV-2, the implications of vertical transmission from mother to baby are still unknown.

The placenta is the interface between maternal and fetal environments that functions to deliver nutrients and oxygen to the fetus. It may also act as a line of defense to limit microbial access to the fetus. However, the phenomenon of immunothrombosis may interfere with placental blood flow to the fetus [9]. Hemostasis and innate immunity assist in the role of recognition and removal of unknown pathogens. However, in inflammatory conditions such as COVID-19, this can result in high rates of thrombosis preventing adequate perfusion of the fetus [9].

Vertical transmission of the SARS-CoV-2 in the third trimester is estimated at 3.5% by infant nasopharyngeal swab testing [10]. However, a systematic review by Hessami et al. found perinatal mortality as a consequence of maternal COVID-19 infection a rare event [11]. Of the 2815 studies screened in the systematic review including maternal, fetal, and neonatal mortality cases associated with COVID-19, there were 37 maternal and 12 perinatal deaths. Fetal and neonatal deaths were postulated to be a result of the severity of maternal infection or prematurity. There was no evidence of vertical transmission or positive COVID-19 results among the expired neonates [11].

Herein, we describe four cases in which vertical transmission has been identified and pathologic specimens of the placenta were obtained. This report is intended to provide a community healthcare practice experience with maternal COVID-19 infection and its consequences in the fetus and neonates.

## Case presentation

All the cases were before the development of the COVID-19 vaccine.

Case 1

The patient, a 22-year-old, gravida 1, para 0, delivered at 36 weeks and six days. The infant weighed 4 pounds 11 ounces with Apgar scores of 8 and 9 at one and five minutes, respectively. Twenty-nine days before delivery, the patient tested positive for COVID-19. She also tested positive for group B *Streptococcus* (GBS).

Gross Examination

The 37-week gestation placenta was small at 340 grams, in the 10th percentile, with extensive villous infarction involving approximately 80% of the placental disc.

Microscopic Examination

The infarction was old with hyalinization and focal disappearance of nuclear material. There was dense perivillous fibrin. At the periphery of the necrotic tissue were residual foci of degenerated intervillous histiocytes, admixed with fibrin. The intervillous space also contained many histiocytic ghost cells. The histologic features suggested resolved massive histiocytic intervillositis. The adjacent viable villous parenchyma showed chorangiosis, consistent with a compensatory response.

By polymerase chain reaction (PCR) analysis, SARS-CoV-2 RNA was detected in placental villous parenchyma. The RNA was detected with a modification of Bio-Rad SARS-CoV-2 ddPCR assay (Bio-Rad Laboratories Inc., Hercules, California, United States) targeting the unique N1 and N2 sequences in the nucleocapsid gene of SARS-CoV-2. The test was performed at Mayo Clinic Laboratories, Rochester, Minnesota. Immunohistochemistry for SARS-CoV-2 spike S1 subunit protein was positive in affected villi, especially in those with less hyalinization.

Case 2

The patient was a 25-year-old female who suffered a second-trimester fetal demise after testing positive for COVID-19.

Gross Examination

The 25th-week gestation placenta was small, weighing 216 grams, less than the 10th percentile.

Microscopic Examination

Villous maturation was appropriate for gestational age. There was extensive histiocytic intervillositis with villous necrosis and perivillous fibrin deposition affecting approximately 50% of villous parenchyma. The intervillous histiocytes stained positive for CD68 and CD163 (CD163 (MRQ-26) Mouse Monoclonal Primary Antibody; F. Hoffmann-La Roche AG, Basel, Switzerland). Immunohistochemistry demonstrated strong staining of syncytiotrophoblasts for SARS-CoV-2 spike S1 subunit protein.

Case 3

The patient, a 29-year-old gravida 4, para 1, abortus 1, presented at 31 weeks and one day gestation with decreased fetal movement. The patient was morbidly obese with a smoking history of seven cigarettes per day. She was febrile with mild upper respiratory symptoms. She was thrombocytopenic with mildly elevated liver transaminases and was diagnosed with preeclampsia-like syndrome. The patient was discharged but returned to the hospital again with decreased fetal movement two days later, after testing positive for COVID-19. She then underwent an emergent C-section. The infant was delivered at 31 weeks and three days gestational age and was in respiratory distress. The infant weighed three pounds 15 ounces at delivery with Apgar scores of 4 and 5 at one and five minutes, respectively.

Gross Examination

**Figure 1:**
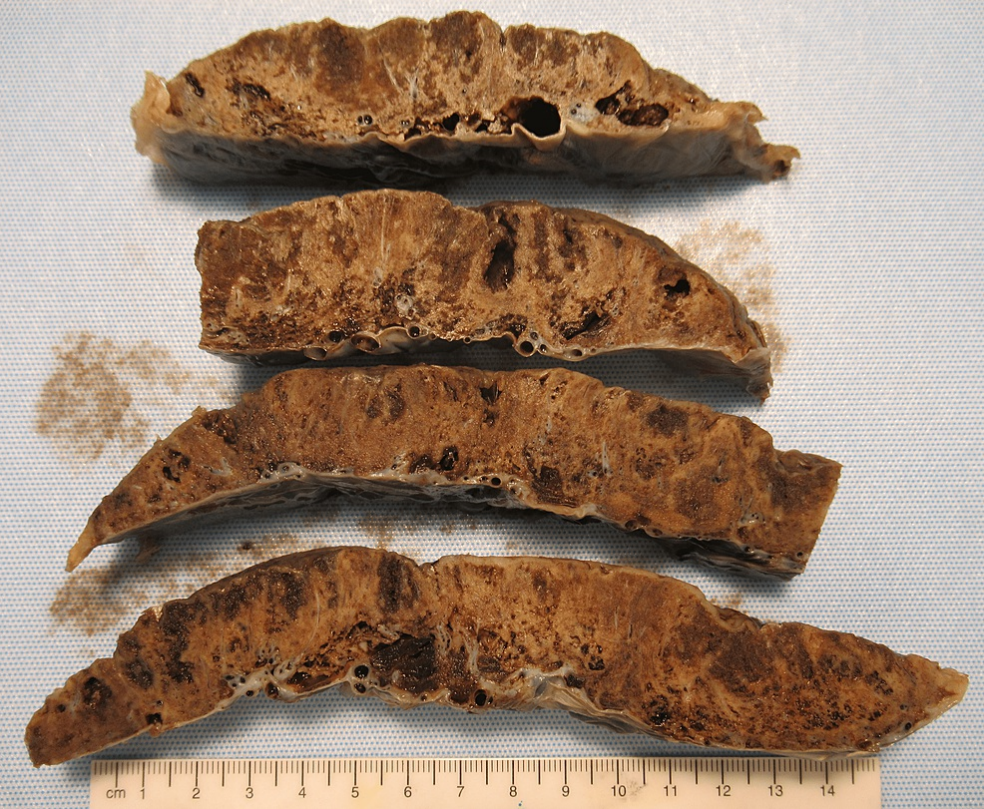
Gross anatomy of placenta, Case 3 Demonstrates abnormal placenta with pale infarcts. The darker areas of the placenta represent normal tissue.

At the time of delivery, the placenta was grossly abnormal, weighing appropriately for gestational age at 352 grams (75th percentile). The parenchyma was firm and pale with splotchy areas of spongy dark normal tissue (Figure 1).

Microscopic Examination

**Figure 2:**
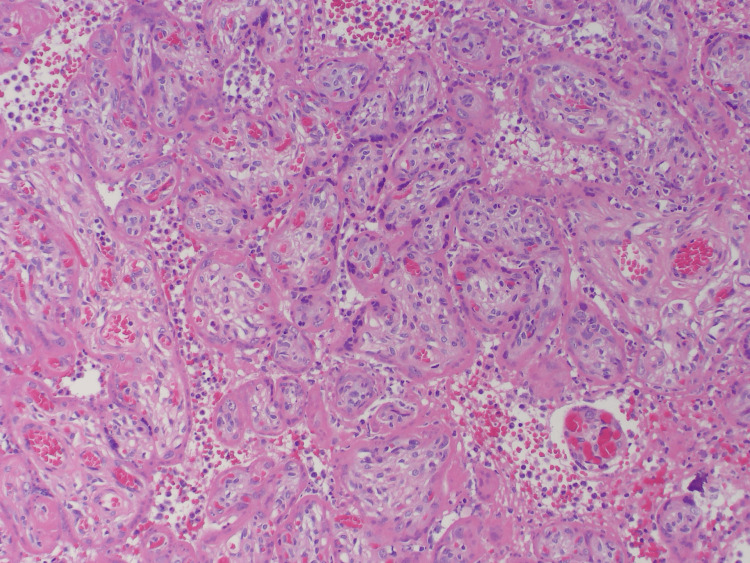
Histology of the pale firm areas of the placenta, Case 3 Coalescing villi is seen in various stages of infarction with obliteration of capillaries, and necrotic syncytiotrophoblasts with disappearing nuclei. There is also perivillous fibrin deposition and stromal fibrosis of the villous parenchyma in later stages of infarction.

**Figure 3:**
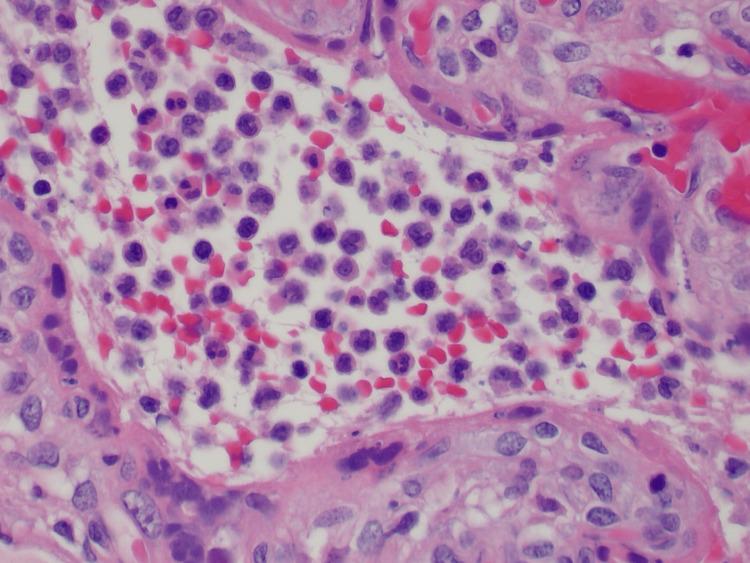
Histiocytic intervillositis with numerous histiocytes with folded nuclear contour, Case 3

Microscopically, the pale firm areas demonstrated coalescing villi in various stages of infarction with obliteration of capillaries, and necrotic syncytiotrophoblasts with disappearing nuclei. In the later stages of infarction, there was perivillous fibrin deposition and stromal fibrosis of the villous parenchyma (Figure 2). There was marked histiocytic intervillositis with numerous histiocytes with folded nuclear contours (Figure 3).

**Figure 4:**
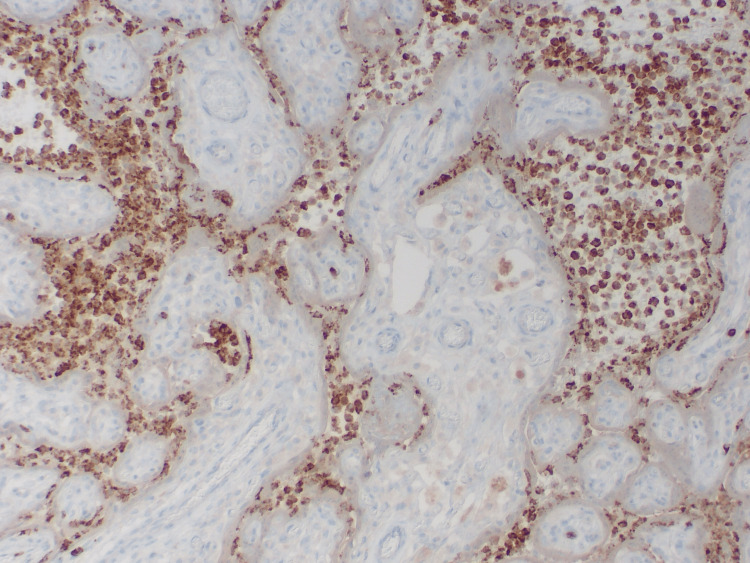
Histiocytes stained positive for CD68, Case 3

**Figure 5:**
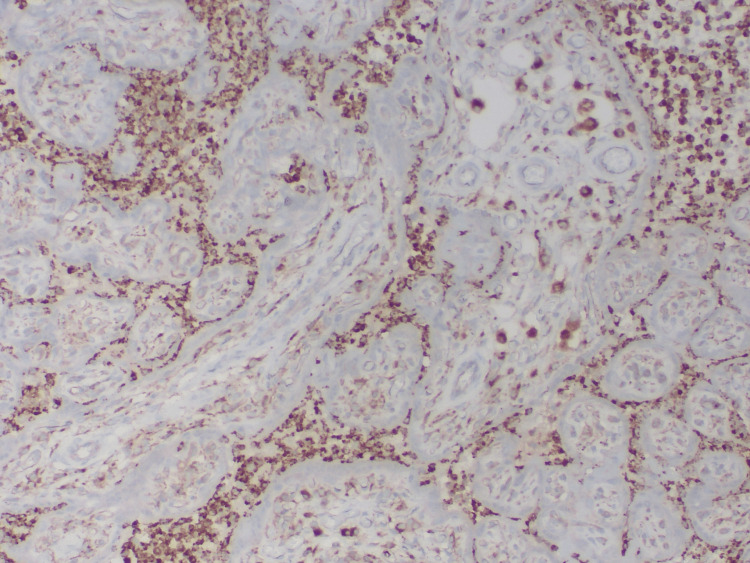
Histiocytes stained positive for lysozyme, Case 3

The histiocytes were positive for CD68 and lysozyme and negative for myeloperoxidase (Figures 4-5). Bacteria were not detected on both Gram and Steiner histochemical stains. Acid-fast microorganisms were not detected on the AFB histochemical stain. Fungal microorganisms were not detected on the Grocott Methenamine Silver (GMS) histochemical stain. There was no evidence of cytomegalovirus (CMV) infection in immunohistochemistry. Epstein-Barr virus was negative by in situ hybridization.

**Figure 6:**
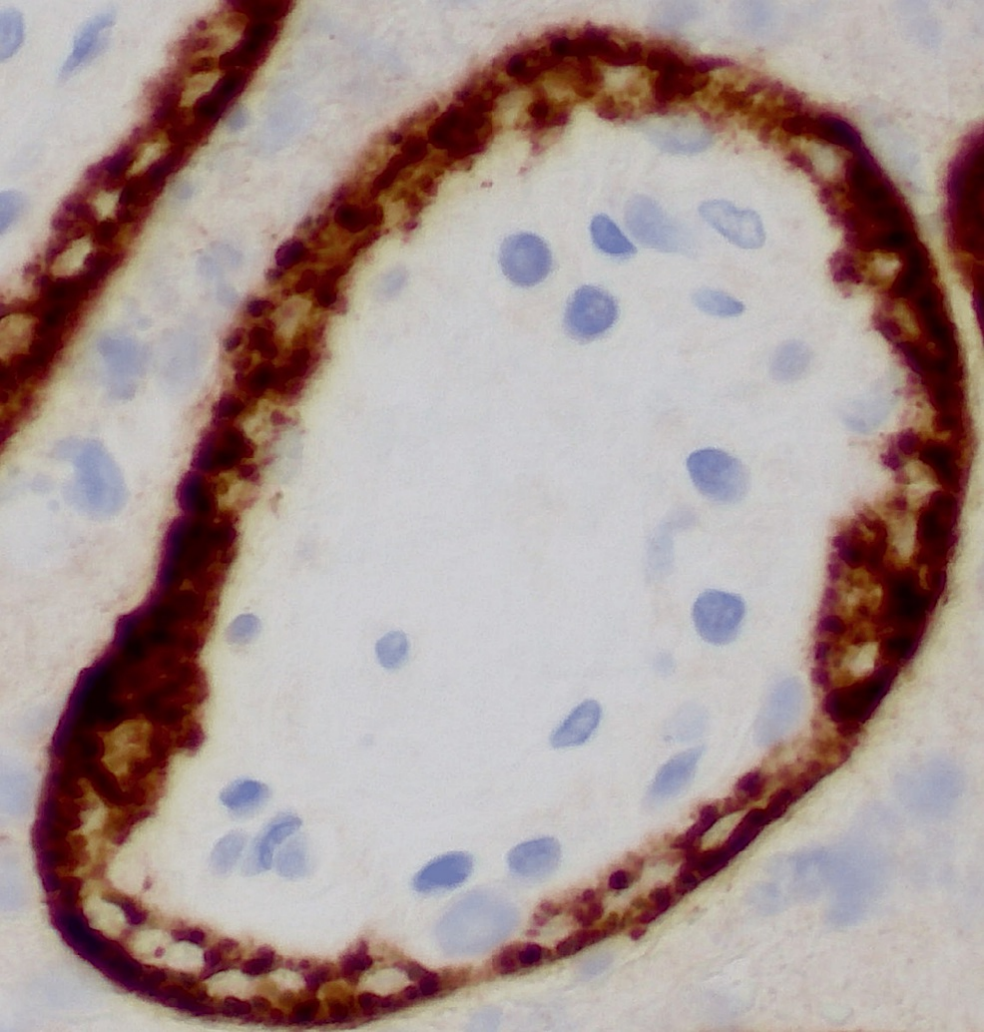
Positive staining with immunohistochemistry with monoclonal antibodies against the SARS-CoV-2 spike S1 subunit protein, Case 3 There is strong cytoplasmic staining within affected syncytiotrophoblasts.

Immunohistochemistry with monoclonal antibodies against the SARS-CoV-2 spike S1 subunit protein showed strong cytoplasmic staining within affected syncytiotrophoblasts (Figure 6). Molecular analysis on paraffin-embedded placental parenchyma using the Droplet Digital™ PCR (ddPCR™) system (Bio-Rad Laboratories, Inc., Hercules, California, United States) for the detection of the N1 and N2 SARS-CoV-2 nuclear capsid genes identified presence of viral RNA within placental tissue. Mass spectrometry on paraffin-embedded placental parenchyma identified the SARS-CoV-2 spike and matrix proteins. Viral RNA was not detected in the neonate's cord blood plasma. Cord blood pH was less than 7, ventilatory assistance was greater than 10 minutes, and there was severe anemia with hematocrit less than 35%.

The infant was transferred for specialized neonatal care. Imaging of the chest showed diffuse ground-glass opacity in the lungs and imaging of the head showed germinal matrix hemorrhage. Two days after delivery, a sample from the nasopharynx of the infant yielded a positive COVID-19 result using the Xpert Xpress SARS-CoV-2 test (Cepheid, California, United States) that detected SARS-CoV-2 RNA. The infant expired that day.

Case 4

The patient was a 27-year-old gravida 2, para 0, abortus 1. The patient presented to the emergency department at 16 weeks and six days of gestation with the complaint of severe lower abdominal cramping and a “gush” of bloody fluid from the vaginal canal, and had a spontaneous vaginal delivery of an expired intact male fetus and placenta shortly after arrival. The patient had tested positive for SARS-CoV-2 six days before presentation and was still symptomatic on presentation. Her symptoms included mild intermittent tachycardia but no respiratory distress. She suffered loss of taste and smell, rhinorrhea, chills, and stated she had a mildly elevated temperature at home, but no fever was measured in the emergency department. The mother’s history was complicated by a prior miscarriage, maternal obesity, and Rh-negative blood type. SARS-CoV-2 RNA was detected in tissue samples of the fetal internal organs and placenta.

Gross Examination

The fetus was normally formed and weighed 103 grams. At 16 weeks gestation, the placenta was immature and diffusely firm with multiple mottled areas throughout. The eyelids were unfused, and the ears appeared normally set. The nose, auditory canals, and anus were probe patent. The external and internal genitalia were phenotypically male. There was an area of disruption on the abdomen superior to the umbilical cord with protrusion of the small intestine through the muscle wall. Internal organs were intact and present with the normal anatomical position.

Microscopic Examination

Prominent histiocytic intervillositis was present with widespread early villous necrosis and perivillous fibrinoid deposition. Histochemical AFB, GMS, Gram, and Steiner staining and immunohistochemistry for CMV were negative for microorganisms. SARS-CoV-2 spike S1 subunit protein was detected by immunohistochemistry. Placental parenchyma tested positive for SARS-CoV-2 RNA. Fetal tissue (internal organs) was carefully dissected to avoid contamination with maternal tissue, and this also tested positive for SARS-CoV-2 RNA.

## Discussion

The coronavirus family has caused several epidemics and pandemics among the human race, with the newest being COVID-19 [12]. SARS-CoV-2 is transmitted primarily via respiratory droplets; however, there is some preliminary evidence for prenatal transmission [13]. The angiotensin-converting enzyme 2 (ACE2) is a receptor present in the alveolar epithelial cells of the lung, to which SARS-CoV-2 binds and enters the host cells [14]. Interestingly, the placenta also expresses the ACE2 receptor after seven weeks gestation, which raises concern for viral entry and fetal infection. The ACE2 receptor has also been isolated in fetal kidney, ilium, and rectal cells as early as 15 weeks gestation [14].

The placenta acts as a protective barrier between mother and fetus [14]. The cells express elevated levels of antimicrobial defensins, toll-like receptors, and nucleotide-binding oligomerization domain proteins [15]. It is speculated that syncytiotrophoblasts have anti-viral qualities via miRNA and type III interferons [15]. Dense, branched microvilli may assist in preventing microbial invasion as well. However, these defenses are not impermeable to all microbes. Studies have confirmed the presence of SARS-CoV-2 mRNA or virions in syncytiotrophoblasts suggesting transplacental infection [14]. The placentas from the current all stained positive for SARS-CoV-2. Additionally, in another study by Facchetti et al., SARS-CoV-2 S and N proteins were strongly expressed in the placenta in mothers infected with the virus [16]. In Case 4 of this report, SARS-CoV-2 RNA was detected in the fetal internal organs as well as the placenta with pronounced histiocytic intervillositis.

Prior to the SARS-CoV-2 pandemic, chorionic histiocytic intervillositis was seen in rare cases, often of unknown etiology. Massive histiocytic intervillositis has emerged as a reliable marker for SAR-CoV-2 infection of the placenta [17]. The inflammatory cells are predominantly histiocytes that stain positive for anti-CD68 [17]; this is also seen in cases 2 and 3 of the present report. Accompanying the histiocytes is often significant perivillous fibrin deposition, with the entrapped villi undergoing cytotrophoblastic cell death, loss of villous capillaries, and eventual villous infarction and fibrosis. Intervillous thrombi, increased microcalcifications, and increased placenta fibrin have been identified in other cases of COVID-19-positive placenta [12,18].

The implications of thrombosis, microcalcifications, and fibrin have been associated with poor obstetric outcomes including intrauterine growth restrictions, preterm delivery, miscarriage, and fetal demise [17,19]. The increase in placental fibrin levels results in decreased maternal placenta blood flow ensuing hypoxic stress in the fetus [18]. Intrauterine hypoxia has been associated with alterations in brain structure and function resulting in defects in motor skills, cerebral palsy, decreased brain weight, schizophrenia, and other forms of cognitive impairment [20]. Furthermore, a higher incidence of severe COVID-19 disease course has been observed in pregnant women. The mRNA vaccines have been found to be effective and safe options to reduce the severity of the infection in pregnant women and fetuses [21].

## Conclusions

Nearly three years after the SARS-CoV-2 epidemic was declared a global pandemic, we have learned much about its complex effects on maternal and fetal health and histopathology changes in COVID-19-infected pregnant women. Yet, there is much more to investigate, including its potential long-term sequelae for neonates with intrauterine infection and preventative measures by improvement of vaccine development. The findings in this report illustrate the pathologic diversity and severity of SARS-CoV-2 infection on the placenta as well as the impact on the health of the fetuses and neonates. With the myriad of patient symptoms and placental pathology, it is currently challenging to predict the outcome of the viral illness.
